# Identification of a novel hypovirulence-inducing ourmia-like mycovirus from *Fusarium solani* causing ginseng (*Panax ginseng*) root rot

**DOI:** 10.3389/fmicb.2025.1609431

**Published:** 2025-07-02

**Authors:** Kaige Ma, Hechi Ni, Zihang Liu, Liping Cai, E. Jiang, Baohui Lu, Lina Yang, Yanjing Zhang, Jie Gao

**Affiliations:** ^1^College of Plant Protection, Jilin Agricultural University, Changchun, China; ^2^State-Local Joint Engineering Research Center of Ginseng Breeding and Application, Changchun, China

**Keywords:** mycovirus, hypovirulence, ourmia-like virus, *Fusarium solani*, ginseng root rot

## Abstract

**Introduction:**

*Fusarium solani* is a widespread plant pathogen known to damage numerous crops, including causing severe root rot in *Panax ginseng*. In this study, we identified a novel ourmia-like mycovirus in *F. solani*, named “*Fusarium solani* ourmia-like virus 1” (FsoOLV1). We demonstrated that FsoOLV1 confers hypovirulence in its host *F. solani* and three other ginseng root rot pathogens, *F. oxysporum*, *F. proliferatum*, and *F. verticillioides*. Additionally, we verified its horizontal and vertical transmission capabilities.

**Methods:**

FsoOLV1 was discovered in *F. solani* strain SJH 2-4 using high-throughput sequencing. The genome sequence of FsoOLV1 was obtained through RT-PCR and RACE. Virus elimination was conducted to assess the effect of FsoOLV1 on fungal virulence. Protoplast transfection experiments were performed to evaluate the impact of the virus on other ginseng root rot pathogens. Horizontal and vertical transmission studies were also carried out to examine the spread of the virus.

**Results:**

The genome of FsoOLV1 is 2,801 nucleotides (nt) in length with a GC content of 47.05%. It encodes a 750 amino acid RNA-dependent RNA polymerase (RdRp), with a molecular weight of approximately 84.84 kDa. Phylogenetic analysis indicated that FsoOLV1 clusters within a clade containing the *Magoulivirus* genus in the *Botourmiaviridae* family. Curing FsoOLV1 from the fungal host strain revealed that the mycovirus plays a role in reducing the virulence of the *F. solani* strain SJH 2–4. Furthermore, protoplast transfection revealed that FsoOLV1 can significantly reduce the virulence of other ginseng root rot pathogens, including *F. oxysporum*, *F. proliferatum*, and *F. verticillioides*. Additionally, FsoOLV1 is capable of horizontal transmission between *F. solani* strains and vertical transmission to the next generation via conidia.

**Discussion:**

This study presents the first hypovirulent ourmia-like virus, FsoOLV1, which reduces the virulence of *F. solani* and other ginseng root rot pathogens. Our findings suggest that FsoOLV1 could serve as a promising biological agent for controlling ginseng root rot. This research not only expands the diversity of known hypovirulent mycoviruses but also provides a potential candidate for controlling Fusarium diseases in ginseng cultivation.

## Introduction

Mycoviruses, or fungal viruses, are pathogens that infect and replicate within major filamentous fungi, yeasts, and oomycetes (Xie and Jiang, 2014; Ghabrial et al., 2015). The modern era of mycovirology began in 1962 when electron microscopy was used to identify diverse virus particles in the fruiting bodies of diseased *Agaricus bisporus* (Hollings, 1962). Since then, the number of reported mycoviruses has increased, and the advent of high-throughput sequencing technologies in recent years has greatly facilitated mycovirus identification. Mycoviruses exhibit diverse types of genomes, including double-stranded RNA (dsRNA), positive-sense single-stranded RNA (+ ssRNA), negative-sense single-stranded RNA (- ssRNA) and single-stranded DNA (ssDNA) genomes (Hough et al., 2023). According to the International Committee on Taxonomy of Viruses (ICTV),^1^ mycoviruses were classified into 42 officially recognized families (Xie and Jiang, 2024). Most of them have + ssRNA genomes, such as the families *Alphaflexiviridae*, *Barnaviridae*, *Botourmiavirida*, *Deltaflexiviridae*, *Endornaviridae*, *Gammaflexiviridae*, *Hypoviridae*, *Mitoviridae*, and *Narnaviridae* (Kotta-Loizou, 2021; Hough et al., 2023).

Generally, most mycoviruses are cryptic and do not significantly affect their fungal hosts. However, some mycoviruses can reduce the virulence of host fungi, a phenomenon known as “hypovirulence” (Nuss, 2005; Ghabrial and Suzuki, 2009). The potential for hypovirulence-associated mycoviruses to be used as biocontrol agents for crop fungal diseases has been previously explored. For example, *Cryphonectria hypovirus* 1 (CHV1) has been successfully used to control chestnut blight in Europe. *Sclerotiorum sclerotiorum* hypovirulence-associated DNA virus 1 (SsHADV-1), which functions as a plant vaccine, can transform its host from a typical necrotrophic pathogen to a beneficial endophytic fungus (Yu et al., 2010; Yu et al., 2013; Zhang et al., 2020). Additionally, many hypovirulence-associated mycoviruses have been identified in diverse phytopathogenic fungi, such as *Alternaria alternata* hypovirus 1 (AaHV1), *F. graminearum* 1 (FgV1), and *Rhizoctonia solani* endornavirus 1 (RsEV1), which demonstrates that they could be effective biological agents for the control of crop fungal diseases (Li H. et al., 2019; Li P. F. et al., 2019; Zheng et al., 2019).

Ourmiaviruses are well-known plant viruses with genomes comprising three + ssRNA segments coding for an RNA-dependent RNA polymerase (RdRp), a movement protein (MP), and a coat protein (CP) (Turina et al., 2017). Recently, many mycoviruses containing a single RNA segment encoding RdRp have been identified as ourmia-like viruses phylogenetically related to *Ourmiavirus* genus. These ourmia-like viruses typically have genome sizes ranging from 2.5 to 3.0 kb, possess a single + ssRNA segment encoding RdRp, and lack a capsid (Ayllón et al., 2020). Consequently, the ICTV established a new family, *Botourmiaviridae*, that includes these ourmia-like viruses. *Botourmiaviridae* includes 12 viral genera: *Betabotoulivirus*, *Betarhizoulivirus*, *Betascleroulivirus*, *Botoulivirus*, *Deltascleroulivirus*, *Epsilonscleroulivirus*, *Gammascleroulivirus*, *Magoulivirus*, *Ourmiavirus*, *Penoulivirus*, *Rhizoulivirus*, and *Scleroulivirus* (Ayllón et al., 2020; Donaire et al., 2024). Recently, an increasing number of ourmia-like viruses have been reported in various fungi, including *Botrytis* (Donaire et al., 2016), *Botryosphaeria dothidea* (Lian et al., 2021; Song et al., 2023), *Colletotrichum* spp. (Guo et al., 2019; Guo et al., 2022; Xu et al., 2022), *F. oxysporum* (Zhao et al., 2020), *Magnaporthe oryzae* (Illana et al., 2017; Li C. X. et al., 2019; Liu et al., 2020; Zhou et al., 2021), *Phoma matteucciicola* (Zhou et al., 2020), *Phomopsis asparagi* (Zhou et al., 2024), *Sclerotinia sclerotiorum* (Wang et al., 2020), and *Verticillium dahlia* (Gao et al., 2022). Notably, an ourmia-like mycovirus (FoOuLV1) from *F. oxysporum* f. sp. momordicae possesses hypovirulence traits (Zhao et al., 2020).

Ginseng (*Panax ginseng* C. A. Mey.) is an araliaceous herb that is vulnerable to root rot diseases induced *Fusarium* spp. which can result in yield losses up to 30–60% in the field (Ma et al., 2023). The reported pathogens of ginseng root rot are mainly *F. oxysporum* and *F. solani* (Lee, 2004; Wang et al., 2022; Wang et al., 2021; Choi et al., 2024). *F. proliferatum*, *F. verticillioides*, *F. subglutinans*, *F. acuminatum*, *F. cerealis*, *F. redolens*, and *F. equiseti* (Gao et al., 2014; Guan et al., 2014; Wang et al., 2016; Guan et al., 2020; Li et al., 2020; Ma et al., 2023) can also cause ginseng root rot. Fusarium root rot is a soil-borne disease that affects plants at all growth stages, and it is known to significantly hinder the cultivation and productivity of various crops (Rampersad, 2020). *Fusarium solani* is an economically significant phytopathogen that causes root rot and fusarium wilt in numerous plants (Jiao et al., 2015; Qiu et al., 2024). To date, four mycoviruses have been identified within *F. solani*, including *F. solani* virus 1 (FsV1) in *F. solani* f. sp. robiniae, *F. solani* partitivirus 2 (FsPV2) in *F. solani* f. sp. pisi (Nogawa et al., 1996; Osaki et al., 2015), *F. solani* partitivirus 3 (FsPV3) in *F. solani* strain Newher-7 (Ma et al., 2024), and *F. solani* alternavirus 1 (FsAV1) in *F. solani* strain NW-FVA 2572 (Lutz et al., 2022). However, no mycovirus has yet been reported to attenuate the pathogenicity of *F. solani*.

In this study, we identified a new hypovirulent ourmia-like virus in *F. solani*, which is one of the main pathogen causing ginseng root rot, named “*Fusarium solani* ourmia-like virus 1” (FsoOLV1). Furthermore, we demonstrated that FsoOLV1 confers hypovirulence in its host *F. solani* and in three other ginseng root rot pathogens, *F. oxysporum*, *F. proliferatum*, and *F. verticillioides*. Additionally, we verified its horizontal and vertical transmission capabilities.

## Materials and methods

### Fungal isolates

In October 2017, several *Fusarium* strains were isolated from ginseng (*Panax ginseng*) root rot tissues from various locations in Jilin Province, China. These strains included *F. solani* SJH 2-4 from Songjianghe County, Baishan City; *F. solani* TH 7-2 from Tonghua City; *F. oxysporum* FS 7-7-3 from Fusong County, Baishan City; *F. verticillioides* BS 1-7 from Baishan City, and *F. proliferatum* DH 8-9 from Dunhua City. These strains were cultured on potato dextrose agar (PDA) medium (20 g/L glucose, 200 g/L potato, and 15 g/L agar) at 28°C. DNA was extracted using standard DNA extraction kits, and the identity of each strain was confirmed through polymerase chain reaction (PCR) amplification with universal (ITS1 and ITS4) and specific (EF-1 and EF-2) primers (O’Donnell et al., 1998; Bellemain et al., 2010). Phylogenetic trees were constructed, and the classification of each strain was further supported by morphological analysis. Details of all primers used in this study are provided in Supplementary Table S1.

### RNA extraction and RT-PCR detection

For total RNA extraction, each strain was cultured on cellophane membranes placed over PDA for 4–5 days. Approximately 0.1 g of fresh mycelia was harvested, pulverized in liquid nitrogen, and processed using a total RNA extraction kit (TIANGEN, Beijing, China).

Double-stranded RNA (dsRNA) was extracted according to the method described by Zhao et al. (2020), with minor modifications. One g of young mycelium that had been cultivated on PDA for 4 days was flash-frozen in liquid nitrogen and ground into a powder. This powder was then transferred into a 10 mL sterile centrifuge tube, to which 2 mL of GPS buffer (glycine 15.0 g/L, Na_2_HPO_4_ 14.2 g/L, NaCl 35.1 g/L, pH 9.6), 2 mL of phenol (pH 8.0), 2 mL of chloroform isoamyl alcohol (24:1), 100 μL of 10% SDS, and 60 μL of β-mercaptoethanol were added. The mixture was shaken, homogenized, and incubated on ice for 30 min. Following centrifugation at 12,000 rpm for 10 min at 4°C, the supernatant was transferred to a new tube. Anhydrous ethanol was added to achieve a final ethanol concentration of 16%, followed by 0.1 g of cellulose powder CF-11 (Sigma Aldrich, St. Louis, MO, United States); it was then mixed and left to stand in an ice bath for 30 min. The dsRNA was precipitated using isopropyl alcohol and sodium acetate, followed by washing with 75% ethanol, and was ultimately dissolved in 30 μL of RNase-free water. This dsRNA was then treated with enzymes including S1 Nuclease and Recombinant DNase I (TaKaRa, Dalian, China) before electrophoresis on a 1.0% agarose gel.

For RT-PCR detection, first-strand cDNA was synthesized using GoScript Reverse Transcriptase (Promega, United States), followed by PCR amplification with specific primers (2-4F and 2-4R), as detailed in Supplementary Table S1.

### cDNA cloning and sequencing

A cDNA library was constructed using the TruSeq™ RNA Sample Preparation Kit, following the manufacturer’s guidelines. Paired-end sequencing of the barcoded library was then performed on the Illumina HiSeq X Ten platform at Shanghai Biotechnology Co., Ltd. Rapid amplification of cDNA ends (RACE) was used to obtain the 5′ and 3′ ends of the viral RNA sequence. The desired PCR products were purified, cloned into the pMD18-T vector (TaKaRa, Dalian, China), and verified by PCR using specific primers (M13-47, M13-48, and RACE primers). Plasmid extraction was then performed, followed by sequencing at Sangon Biotech. The primers used for cDNA cloning and sequencing are provided in Supplementary Table S1. Secondary structure predictions of the 3′- and 5′-termini of the viral RNA were conducted using the UNAfold web server^2^.

### Sequence and phylogenetic analysis

The complete cDNA sequences of viruses present in the *F. solani* strain SJH 2-4 were retrieved from the NCBI GenBank databases and compared with previously reported viral sequences. Potential open reading frames (ORFs) and associated proteins were predicted using DNAMAN 7.0 software and analyzed using the Motif Search Tool^3^. Amino acid sequence alignments were performed using Clustal X software to ensure accuracy and consistency. Phylogenetic relationships based on viral RdRp sequences were analyzed using MEGA 7.0 software and the maximum likelihood method.

### Virus elimination via protoplast regeneration

Protoplast regeneration was employed to remove FsoOLV1 following the method of Wang et al. (2020) with minor modifications. Protoplasts from strain SJH 2-4 were prepared and plated on RM medium, followed by incubation at 28°C. Mycelial tips that emerged were subsequently transferred to PDA medium supplemented with 0.3 mg/mL ribavirin for 10 generations of continuous culture. Total RNA was extracted from the transformants, and the elimination of the virus was verified using RT-PCR with virus-specific primers.

### Viral transmission assay

To assess the vertical transmission of FsoOLV1, 21 individual spores were isolated from the parent strain SJH 2-4 using single-conidium isolation. The conidia were cultured on PDA for 5 d, and RT-PCR was performed to detect FsoOLV1, using primers 2-4F/R (Supplementary Table S1), which amplified a 763-bp product. For horizontal transmission, co-cultivation experiments were conducted. SJH 2-4 served as the donor strain, and TH 7-2 and 2-4 VF1 were used as recipient strains. The strains were co-cultured on PDA plates until the colonies intersected, and mycelial blocks from TH 7-2 and 2-4 VF1 were transferred for further culturing and purification. The presence of FsoOLV1 was confirmed by RT-PCR in the purified strains.

### Viral RNA transfection

PEG-mediated transfection was used for viral RNA transfection (Yu et al., 2010). Protoplasts from *F. oxysporum* strain FS 7-7-3, *F. verticillioides* strain BS 1-7, *F. proliferatum* strain DH 8-9, and *F. solani* strain TH 7-2 were prepared and mixed with total RNA from *F. solani* strain SJH 2-4. The mixture was incubated with PEG buffer (PEG4000 600 g/L, Tris-HCl 50 mM/L, CaCl_2_ 50 mM/L, pH 7.5), spread on RM medium, and incubated at 28°C to facilitate growth. Once transformants emerged, they were subcultured onto PDA medium and subjected to RT-PCR for viral transmission assessment.

### Effects of FsoOLV1 on *Fusarium* spp.

The colony morphology and mycelial diameter of the strains were estimated on PDA plates after 3, 5, and 7 days of inoculation using the 9 mm mycelial plugs. To assess the effect of the virus on mycelial biomass, the mycelia cultured on PDA were scraped off, dried at 80°C for 1 h, and weighed to determine mycelial biomass. The influence of the virus on hyphal morphology was examined by placing hyphal plugs on Spezieller Nährstoffarmer Agar (SNA) medium (KH_2_PO_4_ 1 g/L, KNO_3_ 1 g/L, MgSO_4_⋅7H_2_O 0.5 g/L, glucose 0.2 g/L, sucrose 0.2 g/L, agar powder 20 g/L) in Petri dishes, followed by incubation at 25°C for 10 d. The morphology of the hyphal tips was observed under a microscope. To investigate the effect of the virus on conidia production and morphology, mycelial plugs (9 mm in diameter) were taken from the periphery of 3-day-old colonies on PDA plates, with two plugs per strain, and inoculated into a medium containing 100 mL of carboxymethylcellulose sodium (CMC) (carboxymethylcellulose sodium 15 g/L, NH_4_NO_3_ 1 g/L, NH_2_PO_4_ 1 g/L, MgSO_4_∙7H_2_O 0.5 g/L, and yeast 1 g/L) within a 500 mL flask. This procedure was replicated three times for each strain. The flasks were then placed in a shaker incubator at 25°C (180 rpm). After 3, 5, and 7 days of culture, the liquid medium was filtered using three layers of sterile wipes. The conidia count for each strain was conducted using a hemocytometer, and spore morphology was examined under a microscope; date from 7 days were used for analysis. To evaluate the effect of the virus on spore germination rates, the spore suspensions from each strain cultured in CMC were diluted to a uniform concentration (1 × 10^7^), and 1 mL of this conidial suspension was transferred to 100 mL of YEPD (yeast 3 g/L, peptone 10 g/L, glucose 20 g/L). After incubation at 200 rpm and 25°C for 10 h, the germination rate was assessed under a microscope, and spore morphology was observed. The experiments were independently replicated three times. Data analyses were conducted using SPSS software (version 20.0, IBM Corp.). A one-way analysis of variance (ANOVA) was employed to compare the datasets, followed by Tukey’s honestly significant difference (HSD) test for *post hoc* analysis. The threshold for statistical significance was *P* < 0.05.

### Virulence assay

The virulence of the fungal strains was evaluated through direct inoculation on *Panax ginseng* roots. Healthy, 3-year-old *Panax ginseng* “Damaya” roots were used, and three 1 mm deep holes were created using an inoculation needle. Fungal plugs were then placed onto the wounds, and observations and photographs were taken after 7 days. Control *Panax ginseng* roots were inoculated with pure PDA medium. Lesion sizes were measured at 7 days post-inoculation. Three roots from each group were selected for the experiment, and three replication of this experiment were performed. Statistical analysis was performed using the same method as described previously.

## Results

### Viruses in *F. solani* strain SJH 2-4

Total RNAs were extracted from six *F. oxysporum* strains, one *F. solani* strain, and one *F. proliferatum* strain, all isolated from ginseng (*Panax ginseng*) root rot samples, and subsequently pooled for next-generation sequencing. Nine viral contigs were identified. Using BLASTX on NCBI, one of these viral contigs, contig 30, showed similarity to *Plasmopara viticola* lesion-associated ourmia-like virus 13 (QGY72543). RT-PCR analysis confirmed the presence of contig 30 in the *F. solani* strain SJH 2-4, which was isolated from Songjianghe city in Jilin Province, China. Further RT-PCR detection of nine viral contigs revealed that SJH 2-4 contains only contig 30. Based on these findings, we named this mycovirus as “*Fusarium solani* ourmia-like virus 1” (FsoOLV1).

**FIGURE 1:**
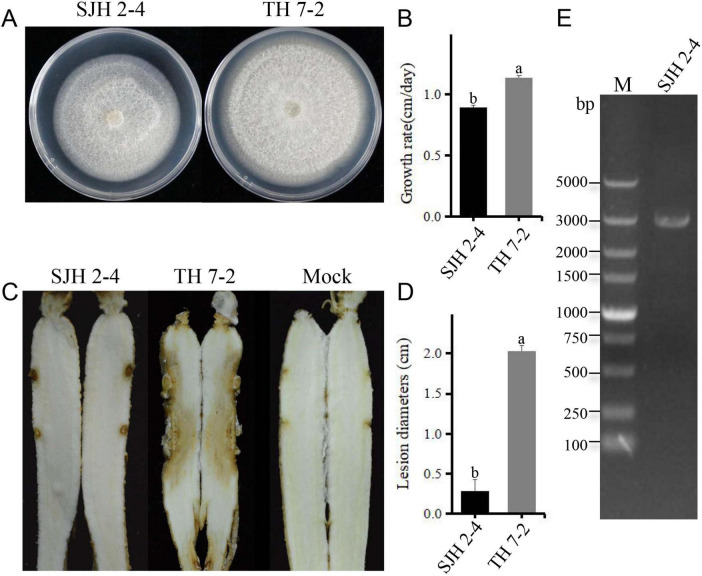
Biological characteristics and dsRNA profile of *Fusarium solani* strain SJH 2-4. **(A)** Colony morphology of *F. solani* strain SJH 2-4 compared with the virulent strain TH 7-2 (cultured on PDA for 5 days at 28°C). **(B)** Growth rate comparison between strains SJH 2-4 and TH 7-2. **(C)** Pathogenicity of strains SJH 2-4 and TH 7-2 on ginseng roots (7 days post-inoculation). The mock group served as a blank control. **(D)** Lesion diameters caused by strains SJH 2-4 and TH 7-2. Error bars represent the standard deviation (SD) of the mean. Different lowercase letters above the columns indicate significant differences (*p* < 0.05). **(E)** Agarose gel electrophoresis of the dsRNA extracted from strain SJH 2-4, treated with DNase I and S1 nuclease. M, molecular weight marker.

Although the colony morphology of SJH 2-4 on PDA medium was similar to that of the virus-free *F. solani* strain TH 7-2, its growth rate was significantly slower (Figures 1A, B). Virulence assessment revealed that SJH 2-4 caused small lesions on *Panax ginseng* roots, in contrast to the severe root rot caused by the virus-free TH 7-2 strain (Figures 1C, D). Additionally, the fragment size of dsRNA extracted from strain SJH 2-4 and treated with DNase I and S1 nuclease was approximately 3.0 kb (Figure 1E).

### Molecular characterization of FsoOLV1

**FIGURE 2:**
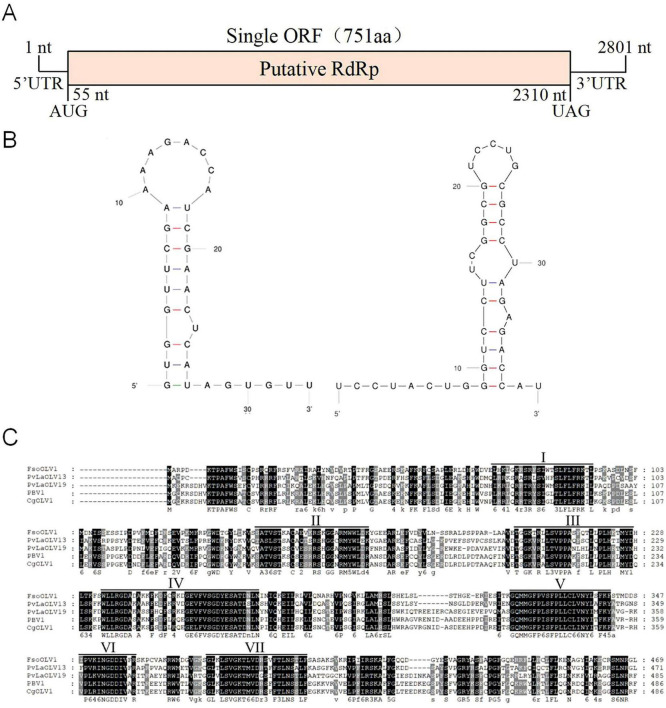
Genomic features of *Fusarium solani* ourmia-like virus 1 (FsoOLV1). **(A)** Schematic representation of the genomic organization of FsoOLV1. The open reading frame (ORF) encoding the RNA-dependent RNA polymerase (RdRp) domain is depicted as a pink box (55–2,310 nt). **(B)** Potential secondary structures of the 5’- (left) and 3’- (right) termini of FsoOLV1. **(C)** Multiple sequence alignment of the amino acid sequences of RdRp proteins encoded by FsoOLV1 and other selected ourmia-like viruses. Seven conserved RdRp motifs are indicated with Roman numerals I to VII. Abbreviations of the viruses are as follows: FsoOLV1, *Fusarium solani* ourmia-like virus 1; PvlaOLV 13, *Plasmopara viticola* lesion associated ourmia-like virus 13; PvlaOLV 19, *Plasmopara viticola* lesion associated ourmia-like virus 19; PBV1, *Pestalotiopsis* botourmiavirus 1; CgOLV 1, *Colletotrichum gloeosporioides* ourmia-like virus 1.

The full-length cDNA sequences of FsoOLV1 were determined by cloning and assembling cDNAs obtained from RT-PCR and RACE (Supplementary Figure S1). The complete sequence of FsoOLV1 spans 2,801 nucleotides (nt) and was submitted to GenBank under accession number OP807121. The genome of FsoOLV1 has a GC content of 47.05% and contains a single ORF that encodes an RdRp consisting of 750 amino acids, with a molecular weight of approximately 84.84 kDa. This ORF starts at an AUG start codon at position 55 and ends at a UAG stop codon at position 2,310 (Figure 2A). The terminal stem-loop structure of FsoOLV1 was predicted using the UNAfold web server. The results showed that the first 33 nucleotides at the 5′ terminus could form a stable stem-loop structure, while the final 39 nucleotides at the 3′ terminus formed two stable stem-loop structures (Figure 2B), which is a distinctive feature of ourmia-like viruses.

Homology searches using BLASTP revealed that the protein encoded by the ORF shares a conserved domain closely related to the RdRps of *Plasmopara viticola* lesion-associated ourmia-like virus 13 (YP_010800213) and *Plasmopara viticola* lesion-associated ourmia-like virus 19 (YP_010800219), with amino acid identities of 49.03% and 43.77%, respectively. Multiple sequence alignments performed using GeneDoc software indicated that the RdRp of FsoOLV1 contains seven conserved motifs, which further clarifies its genetic structure and relationships with other ourmia-like viruses (Figure 2C).

### Phylogenetic analysis of FsoOLV1

**FIGURE 3:**
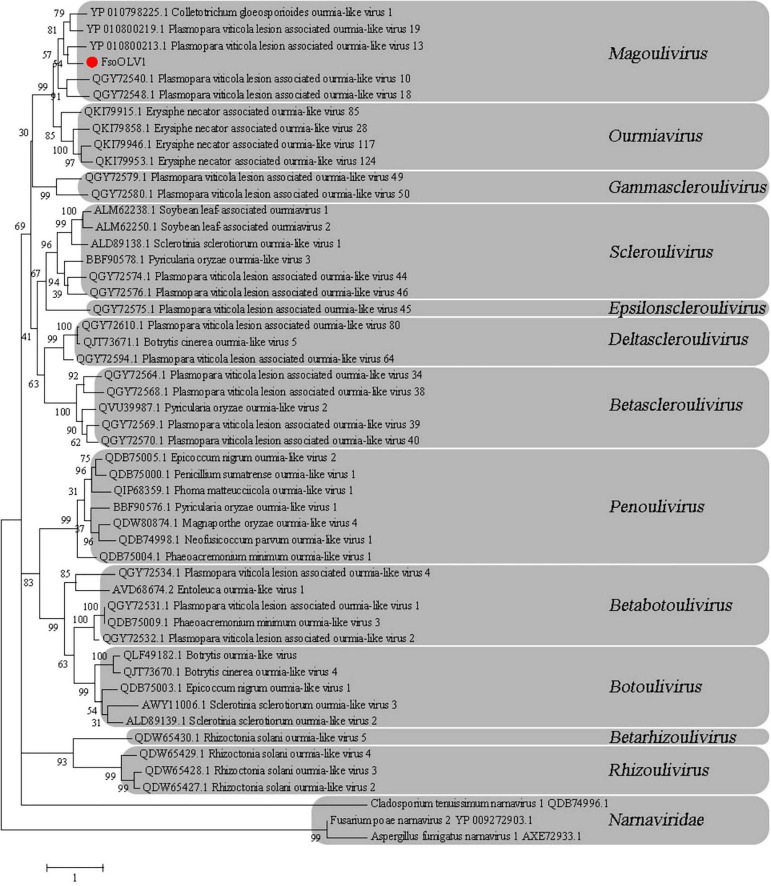
Phylogenetic analysis of FsoOLV1 and other related viruses based on RdRp sequences. Phylogenetic tree constructed using the maximum likelihood method with 1,000 bootstrap replicates. The virus studied in this paper is marked with a red dot. The scale bar represents a genetic distance of 1.

To investigate the phylogenetic relationship between FsoOLV1 and other mycoviruses, a phylogenetic tree was constructed using the maximum likelihood method (ML) based on the RdRp amino acid sequences of FsoOLV1 and related viruses in the *Botourmiaviridae* family, including *Betascleroulivirus*, *Deltascleroulivirus*, *Gammascleroulivirus*, *Epsilonscleroulivirus*, *Ourmiavirus*, *Magoulivirus*, *Betabotoulivirus*, *Scleroulivirus*, *Betarhizoulivirus*, *Botoulivirus*, *Penoulivirus*, and *Rhizoulivirus*, as well as viruses from the *Narnarviridae* family. FsoOLV1 shares a close phylogenetic relationship with the genus *Magoulivirus* and was grouped in the same clade as *Plasmopara viticola* lesion-associated ourmia-like virus 13 (Figure 3). Based on these results, FsoOLV1 was classified as a member of the genus *Magoulivirus* within the family *Botourmiaviridae*.

### Effects of FsoOLV1 on the growth and pathogenicity of *F. solani*

**FIGURE 4:**
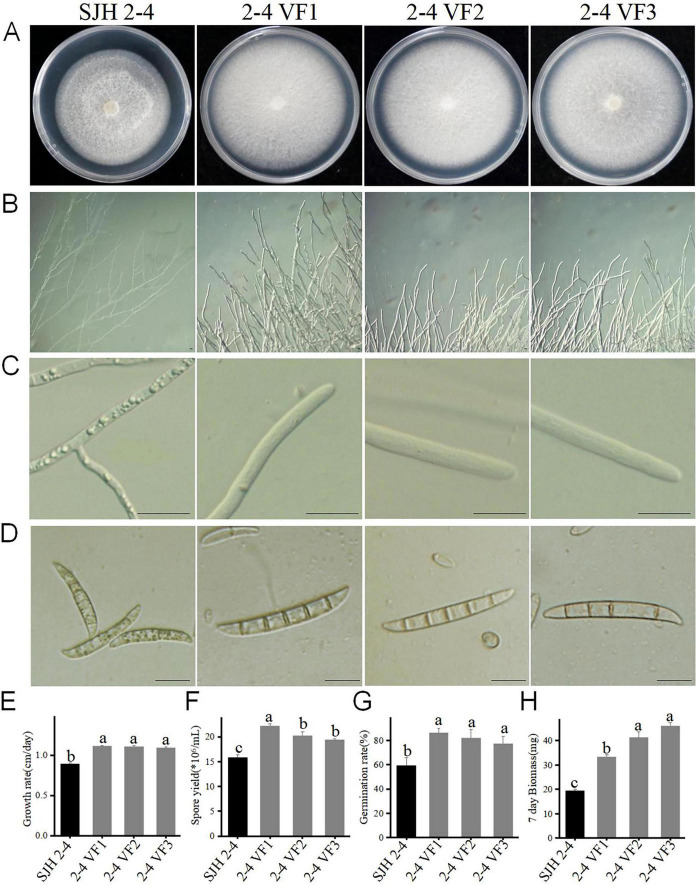
Effect of FsoOLV1 on the biological characteristics of *Fusarium solani* strain SJH 2-4. Colony morphology **(A)**, hyphal tip morphology **(B)**, detailed view of hyphal tips **(C)**, and macrocondium morphology **(D)** of the original strain SJH 2-4 and three virus-free strains (2-4 VF1-3). Scale bars = 20 μm. Comparison of the mycelial growth rate **(E)**, spore yield **(F)**, spore germination rate **(G)**, and mycelial biomass **(H)** of SJH 2-4 and the virus-free strains. Error bars represent the standard deviation (SD) of the means. Different lowercase letters above columns indicate significant differences (*p* < 0.05).

Pathogenicity testing of the FsoOLV1-containing strain SJH 2-4 and virus-free strain TH 7-2 on *Panax ginseng* roots revealed that FsoOLV1 reduced the virulence of *F. solani*, as previously described (Figure 1C). To further investigate the effects of FsoOLV1 on the growth and pathogenicity of *F. solani*, isogenic FsoOLV1-free strains, designated 2-4 VF 1-7, were derived from SJH 2-4 through protoplast regeneration (Supplementary Figure S2). Three of these virus-free strains, 2-4 VF1, 2-4 VF2, and 2-4 VF3, were selected for further study. When cultured on PDA medium for 7 days, the virus-free strains exhibited a faster growth rate compared with SJH 2-4 (Figures 4A, E). The hyphae of SJH 2-4 were irregular, slender, curved, and short-branched when cultured on SNA medium for 10 days, whereas the hyphae of the virus-free strains 2-4 VF1, 2-4 VF2, and 2-4 VF3 were neat and regular (Figure 4B). Additionally, SJH 2-4 has abnormally large vacuoles in its hyphal cells on SNA medium, a feature not observed in the three virus-free strains (Figure 4C). Similarly, after 5 days in CMC medium, SJH 2-4 exhibited malformed and shrunken macroconidia that were filled with numerous vacuoles, while the macroconidia of the virus-free strains appeared regular and healthy (Figure 4D). Spore production and mycelial biomass measurements showed that the virus-free strains 2-4 VF1, 2-4 VF2, and 2-4 VF3 had significantly higher conidial yields and mycelial biomass compared with the original SJH 2-4 strain (Figures 4F, H; Supplementary Table S2). The spore germination rate of the three virus-free strains ranged from 77.47% to 86.65%, which was notably higher than the 59.71% observed for strain SJH 2-4 (Figure 4G; Supplementary Table S2). Collectively, these results suggest that FsoOLV1 significantly affects the fungal host by altering the hyphal growth rate, mycelial biomass, hyphal and spore morphology, and spore germination rate.

**FIGURE 5:**
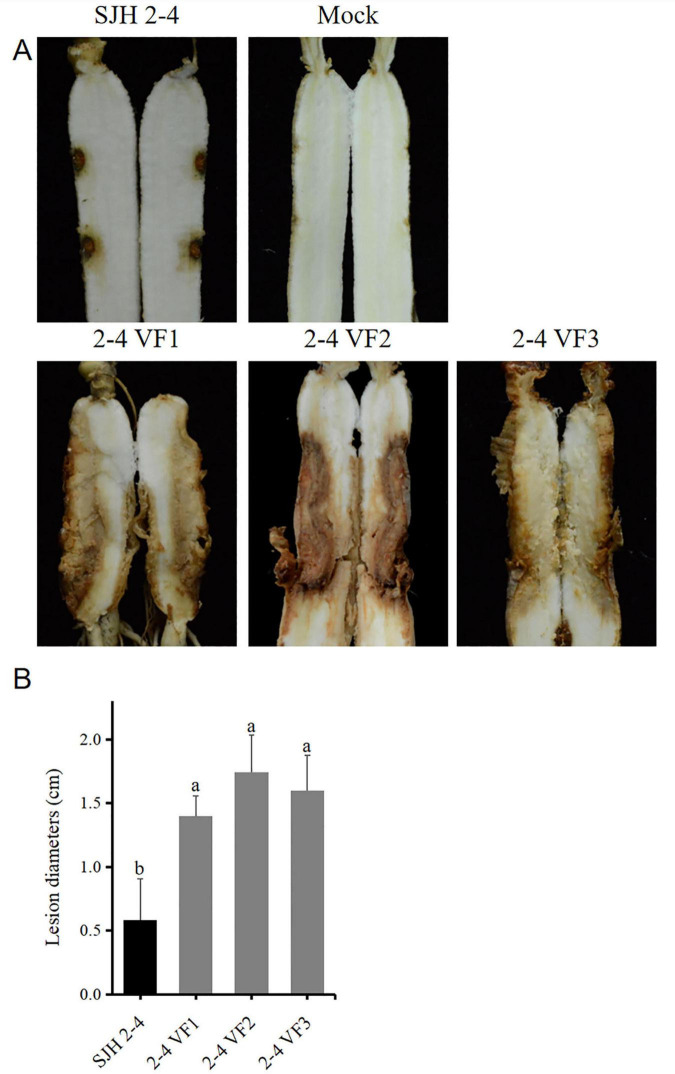
Pathogenicity detection of *Fusarium solani* strain SJH 2-4 and virus-free strains (2-4 VF1-3). **(A)** Symptoms observed on ginseng roots inoculated with the original strain SJH 2-4 and three virus-free strains. The mock group served as a blank control. **(B)** Lesion diameters induced on ginseng roots. Error bars represent the standard deviation (SD) of the mean. Different lowercase letters above columns indicate significant differences (*p* < 0.05).

To evaluate the effect of FsoOLV1 on the virulence of its host fungus, SJH 2-4 and the virus-free strains were tested on *Panax ginseng* roots. Seven days after fungal inoculation, the three virus-free strains led to severe root rot, while SJH 2-4 only resulted in small disease lesions (Figure 5A). The lesion areas caused by the three virus-free strains were notably larger than those caused by SJH 2-4 (Figure 5B). These observations indicate that FsoOLV1 is responsible for the hypovirulence of *F. solani* strain SJH 2-4.

### Transmission of FsoOLV1

**FIGURE 6:**
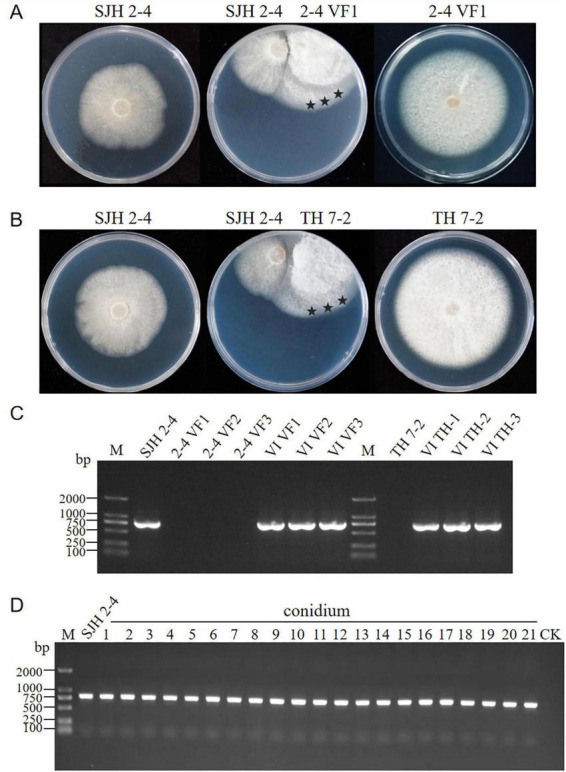
Horizontal and vertical transmission of FsoOLV1. **(A,B)** Horizontal transmission of FsoOLV1 from the donor strain SJH 2-4 to the recipient strains 2-4 VF **(A)** and TH7-2 **(B)** via hyphal anastomosis. Stars represent the regions where mycelial plugs were taken and cultured for subsequent RT-PCR detection. **(C)** RT-PCR detection of FsoOLV1 in the recipient strains using virus-specific primers. **(D)** Vertical transmission of FsoOLV1. Single conidial sub-isolates from the original strain SJH 2-4 were tested for FsoOLV1 presence using RT-PCR.

To assess the horizontal transmission capabilities of FsoOLV1, dual-culture assays were conducted on PDA medium using the FsoOLV1-infected donor strain SJH 2-4 and two virus-free recipient strains, 2-4 VF1 and TH 7-2, respectively. After 5 days of co-cultivation, mycelial plugs were taken from three distinct regions on recipient sides and transferred to fresh PDA plates, yielding in six derived strains: VI VF1, VI VF2, VI VF3, VI TH-1, VI TH-2, and VI TH-3 (Figures 6A, B). RT-PCR analysis confirmed successful horizontal transmission of FsoOLV1 from SJH 2-4 to both recipient strains 2-4 VF1 and TH 7-2 (Figure 6C). However, efforts to transfer FsoOLV1 to other *Fusarium* species, including *F. oxysporum*, *F. proliferatum*, and *F. verticillioides*, were unsuccessful (data not shown).

To determine whether FsoOLV1 can be vertically transmitted, 21 single-conidium-derived colonies from strain SJH 2-4 were analyzed using RT-PCR with FsoOLV1-specific primers. The results showed that all isolates were FsoOLV1 positive (Figure 6D). Furthermore, no significant differences in growth rates were observed among these isolates when cultured on PDA plates. These findings indicate that FsoOLV1 is stably and efficiently transmitted through *F. solani* conidia.

### Effects of FsoOLV1 in other *Fusarium* species

**FIGURE 7:**
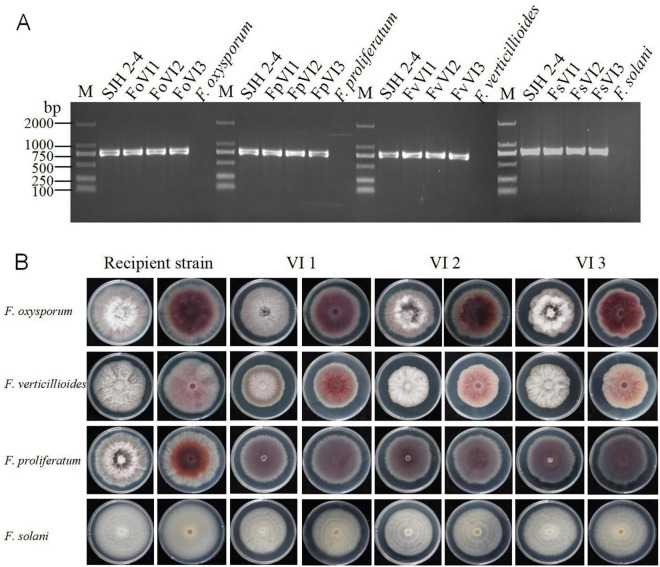
Effects of FsoOLV1 on fungal morphology in four *Fusarium* species. **(A)** RT-PCR detection of FsoOLV1 in four *Fusarium* species using virus-specific primers. M, 2,000 bp DNA marker. **(B)** Colony morphology of the original recipient strains and FsoOLV1-infected (VI) strains after 7 days of culture on PDA at 28°C.

**FIGURE 8:**
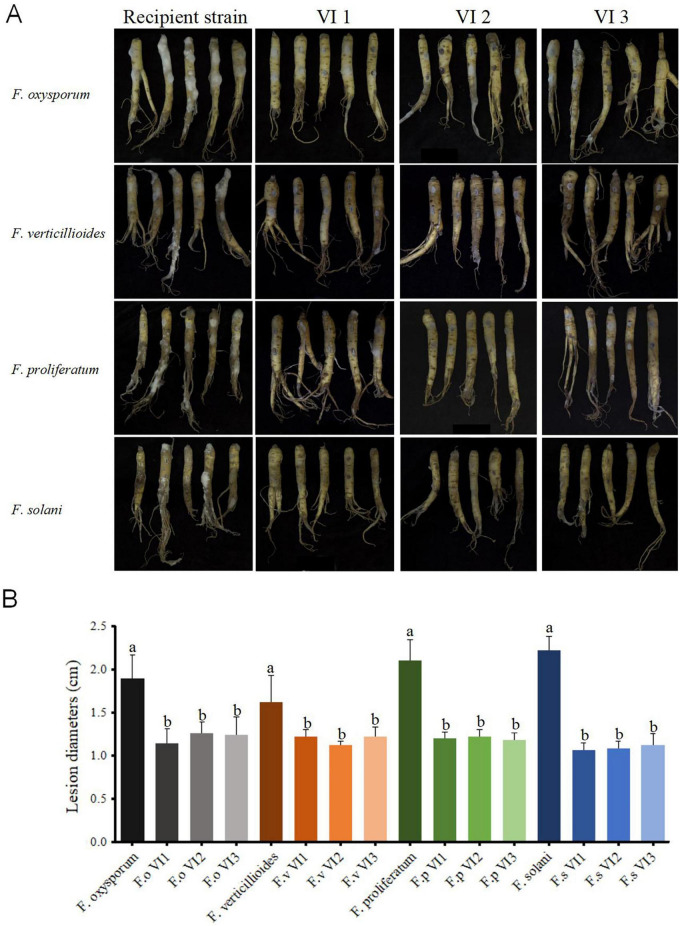
FsoOLV1 induces hypovirulent phenotypes in four *Fusarium* species. **(A)** Virulence assay of four original *Fusarium* species and FsoOLV1-infected (VI) strains on ginseng roots. **(B)** Lesion diameters induced on ginseng roots. Error bars represent the standard deviation (SD) of the means. Different lowercase letters above columns indicate significant differences (*p* < 0.05).

To assess the infectivity of FsoOLV1 in other *Fusarium* species responsible for *Panax ginseng* root rot, FsoOLV1 was transfected to *F. oxysporum*, *F. proliferatum*, and *F. verticillioides* via protoplast transformation. Transformants were obtained from *F. oxysporum* strain FS 7-7-3 (FoVI1, FoVI2, FoVI3), *F. proliferatum* strains DH 8-9 (FpVI1, FpVI2, FpVI3), and *F. verticillioides* strain BS 1-7 (FvVI1, FvVI2, FvVI3), using PEG-mediated protoplast transfection. The control strains, FsVI1, FsVI2, and FsVI3, were derived from *F. solani* strain TH 7-2. RT-PCR detection confirmed the successful transfection of FsoOLV1 into the four *Fusarium* species (Figure 7A). Compared with the original strains, the FsoOLV1 transformants had slower growth rates, reduced spore production, lower spore germination rates, and decreased mycelial biomass (Figure 7B; Supplementary Figure S3; Supplementary Table S3). Microscopic observation revealed that the hyphae of FsoOLV1 transformants were twisted, sparse, and less branched (Supplementary Figure S4). Additionally, numerous vacuoles were observed in the hyphal cells of the FsoOLV1 transformants (Supplementary Figure S5). When *Panax ginseng* roots were inoculated with the original strains, they developed significantly more severe root rot symptoms compared with those inoculated with the FsoOLV1-carrying transformants (Figures 8A, B). These findings indicate that FsoOLV1 can confer hypovirulence in other *Fusarium* species. Based on these results, FsoOLV1 could be used as a biocontrol agent for managing ginseng root rot diseases.

## Discussion

In this study, we identified and characterized a novel ourmia-like mycovirus, FsoOLV1, in *F. solani* causing ginseng (*Panax ginseng*) root rot. Based on BLASTX searches, homologous genome comparison, and phylogenetic analysis, we propose that FsoOLV1 is a novel member of the genus *Magoulivirus* within the family *Botourmiaviridae*. Additionally, virus elimination, virus transfection, and biological comparisons demonstrated that FsoOLV1 can induce hypovirulence in four species of *Fusarium*, including the host *F. solani*. To the best of our knowledge, this study is the first to report a hypovirulence-inducing ourmia-like mycovirus in *F. solani*.

*Fusarium solani* is a plant pathogenic fungus with a wide distribution. It causes Fusarium root rot in various economically important crops and results in serious economic losses. Currently, only a few mycoviruses have been identified in *F. solani*, including *F. solani* virus 1 (FsV1) in *F. solani* f. sp. robiniae, *F. solani* partitivirus 2 (FsPV2) in *F. solani* f. sp. pisi (Nogawa et al., 1996; Osaki et al., 2015), *F. solani* partitivirus 3 (FsPV3) in *F. solani* strain Newher-7 (Ma et al., 2024), and *F. solani* alternavirus 1 (FsAV1) in *F. solani* strain NW-FVA 2572 (Lutz et al., 2022). However, none of these mycoviruses have been shown to influence the virulence of their fungal hosts. FsoOLV1 is the first ourmia-like mycovirus identified to induce hypovirulence in *F. solani*.

To date, ourmia-like viruses have been discovered in various fungal species, including *Botrytis* ourmia-like virus (BOLV) in *Botrytis* (Donaire et al., 2016), *Botryosphaeria dothidea* ourmia-like virus 1 (BdOLV1) and *B. dothidea* ourmia-like virus 2 (BdOLV2) in *B. dothidea* (Lian et al., 2021; Song et al., 2023), *Colletotrichum camelliae* botourmiavirus 1 (CcBV1) in *C. camelliae* (Xu et al., 2022), *C. gloeosporioides* ourmia-like virus 1 (CgOLV1) in *C. gloeosporioides* (Guo et al., 2019), *C. fructicola* ourmia-like virus 2 (CfOLV2) in *C. fructicola* (Guo et al., 2022), *F. oxysporum* ourmia-like virus 1 (FoOuLV1) in *F. oxysporum* (Zhao et al., 2020), multiple ourmia-like viruses in *Magnaporthe oryzae* (Illana et al., 2017; Li P. F. et al., 2019; Liu et al., 2020; Zhou et al., 2021), *Phoma matteucciicola* ourmia-like virus 1 (PmOLV1) in *P. matteucciicola* (Zhou et al., 2020), *Phomopsis asparagi* magoulivirus 1 (PaMV1) in *P. asparagi* (Zhou et al., 2024), *Sclerotinia sclerotiorum* ourmia-like virus 4 (SsOLV4) in *S. sclerotiorum* (Wang et al., 2020), and *Verticillium dahliae* magoulivirus 1 (VdMoV1) in *V. dahlia* (Gao et al., 2022). Most of these ourmia-like viruses have no effect or little effect on the host fungi, although some have been found to reduce the pathogenicity of their hosts. For example, CfOLV2 in *C. fructicola* reduces the growth rate of the host and influences the integrity of the cell wall (Guo et al., 2022). Similarly, FoOuLV1 in *F. oxysporum* f. sp. momordicae (FoM) induces hypovirulence in the host strain FoOuLV1 and can be transmitted horizontally within different *formae speciales* within *F. oxysporum* species, such as *F. oxysporum* f. sp. cucumerinum (Zhao et al., 2020). We found that FsoOLV1 not only attenuates the virulence of *F. solani* but also induces hypovirulence in other *Fusarium* species, indicating that it could be effective for the biological control of this pathogen.

The transmission of mycoviruses is a crucial aspect underlying their success as biological control agents. The traditional view is that mycoviruses are transmitted both vertically via spores and horizontally via hyphal anastomosis. Vegetative incompatibility, which is often controlled by multiple vegetative incompatibility genes, is commonly observed among fungal species. FsoOLV1 can be transmitted via conidial and hyphal contact within *F. solani* but cannot be transmitted to *F. oxysporum*, *F. proliferatum*, and *F. verticillioides*. Previous studies have shown that the horizontal transmission of mycoviruses occurs more frequently under natural field conditions compared with laboratory settings, and plant proline accumulation induced by fungal infection enhances mycovirus transmission (Hai et al., 2024). Additionally, exogenous proline has been shown to promote mycovirus transmission in PDA medium and improve the biocontrol efficiency of mycoviruses (Hai et al., 2024). Further research is needed to investigate whether proline enhances the horizontal transmission rate of FsoOLV1 between different *Fusarium* species.

FsoOLV1 not only induces hypovirulence in *F. solani* but also has a hypovirulent effect in other *Fusarium* species, which underscores its potential for future applications. In future studies, the effect of FsoOLV1 on the pathogenicity of other fungal pathogens will be clarified. Additionally, transcriptome sequencing could be used to identify genes and pathways sensitive to FsoOLV1 and provide further insight into the molecular basis of its hypovirulence-inducing effect. These studies will enhance our understanding of FsoOLV1 and its potential applications in biological control and shed light on the diversity of mycoviruses and their ability to control fungal diseases.

## Data Availability

The sequence file of FsoOLV1 is available in the NCBI, GenBank, Accession No. OP807121.

## Footnotes

1
https://ictv.global/taxonomy

2
https://www.unafold.org/mfold/applications/rna-folding-form.php

3
https://www.genome.jp/tools/motif/
