# Supplementary material for: Identification of a novel hypovirulence-inducing ourmia-like mycovirus from Fusarium solani causing ginseng (Panax ginseng) root rot
Source: Front Microbiol. 2025 Jul 2;16:1609431. doi: 10.3389/fmicb.2025.1609431 (PMC12263584; doi:10.3389/fmicb.2025.1609431)
Supplement: Supplementary file 1 [file Table_1.docx]

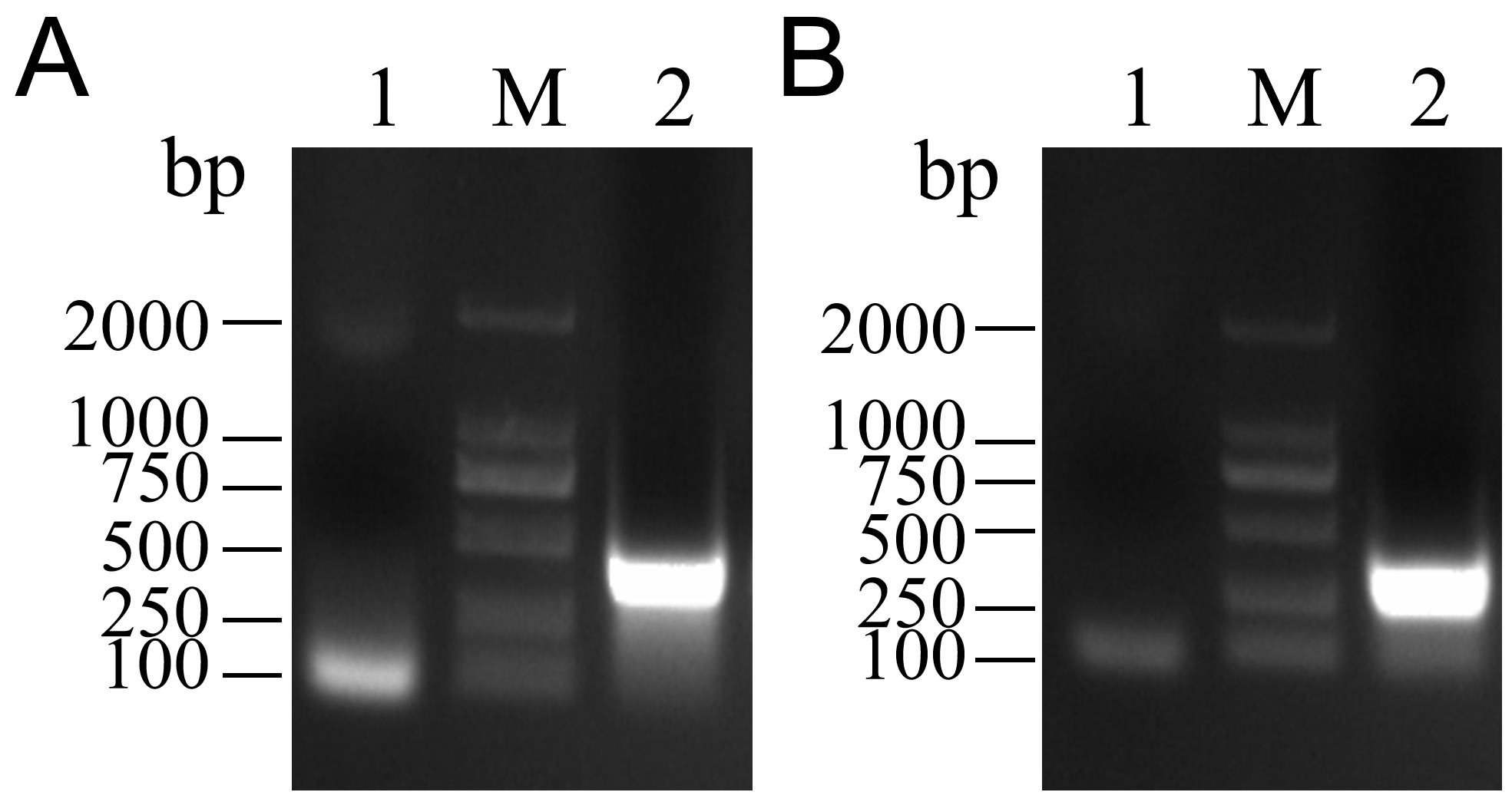


**Figure S1.** Cloning of the termini of the FsoOLV1 genome. (A) 3’RACE. Lane 1: no-template control; Lane M: 2,000 bp DNA marker; Lane 2: 3'-RACE amplification products. (B) 5’RACE: Lane 1: no-template control; Lane M: 2,000 bp DNA marker; Lane 2: 5'-RACE amplification products.
