# Supplementary material for: Identification of a novel hypovirulence-inducing ourmia-like mycovirus from Fusarium solani causing ginseng (Panax ginseng) root rot
Source: Front Microbiol. 2025 Jul 2;16:1609431. doi: 10.3389/fmicb.2025.1609431 (PMC12263584; doi:10.3389/fmicb.2025.1609431)
Supplement: Supplementary file 2 [file Table_2.docx]

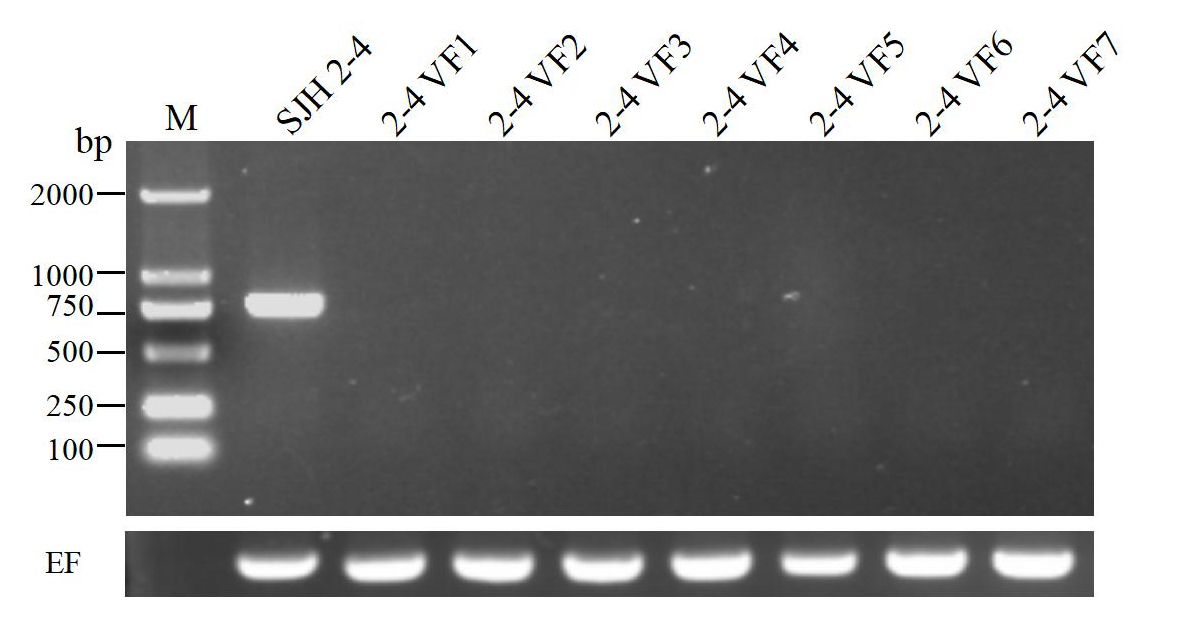


**Figure S2.** RT-PCR detection of FsoOLV1 in *Fusarium solani* SJH 2-4 and its derived curing strains. M, 2000 bp DNA marker; EF represents the reference gene, translation elongation factor 1 alpha.
