# Supplementary material for: Identification of a novel hypovirulence-inducing ourmia-like mycovirus from Fusarium solani causing ginseng (Panax ginseng) root rot
Source: Front Microbiol. 2025 Jul 2;16:1609431. doi: 10.3389/fmicb.2025.1609431 (PMC12263584; doi:10.3389/fmicb.2025.1609431)
Supplement: Supplementary file 3 [file Table_3.docx]

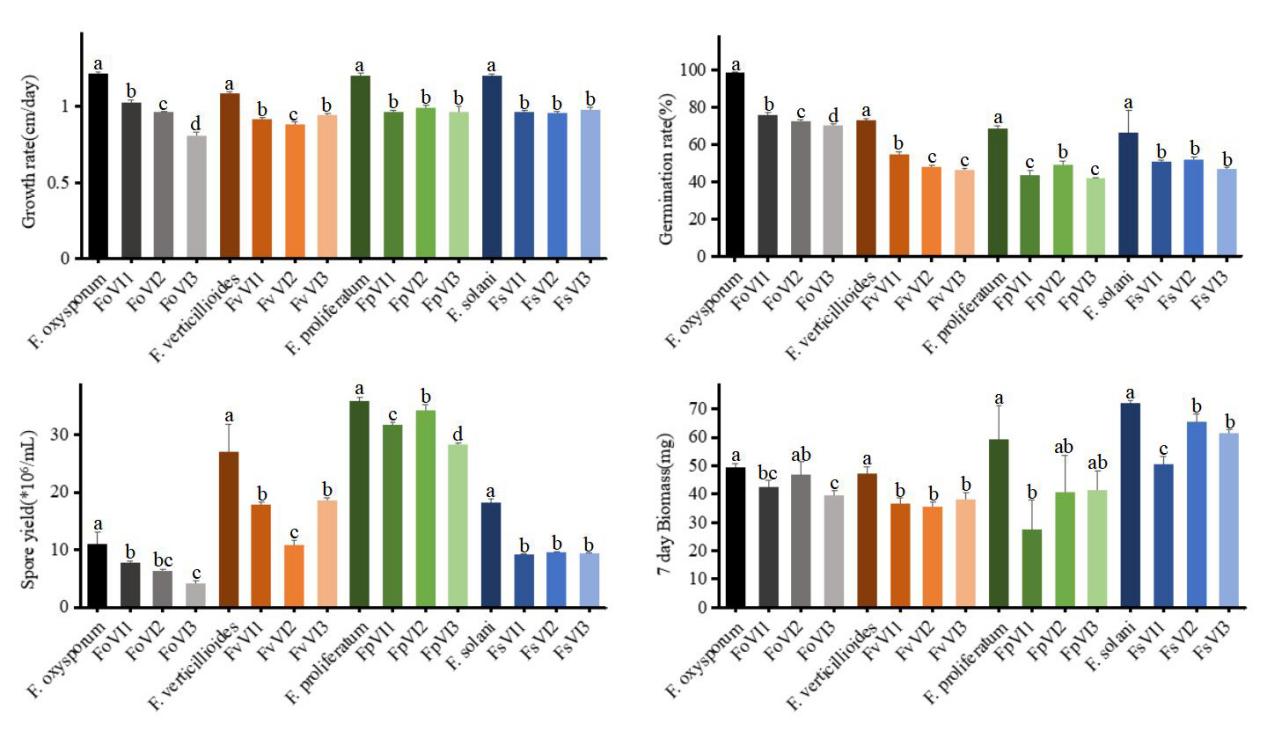


C

D

B

A

**Figure S3.** Biological characteristics of four *Fusarium* species before and after FsoOLV1 transfection.
