# Supplementary material for: Identification of a novel hypovirulence-inducing ourmia-like mycovirus from Fusarium solani causing ginseng (Panax ginseng) root rot
Source: Front Microbiol. 2025 Jul 2;16:1609431. doi: 10.3389/fmicb.2025.1609431 (PMC12263584; doi:10.3389/fmicb.2025.1609431)
Supplement: Supplementary file 4 [file Table_4.docx]

**Recipient strain VI 1 VI 2 VI 3**

***F. oxysporum***

***F. proliferatum***

***F. verticillioides***

***F. solani***


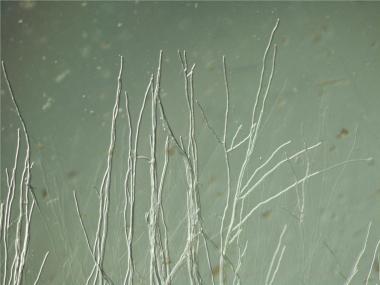

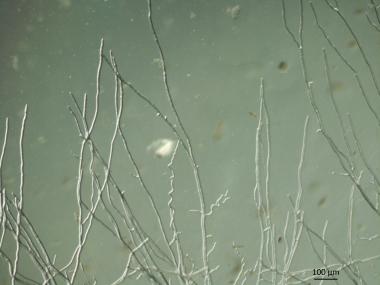

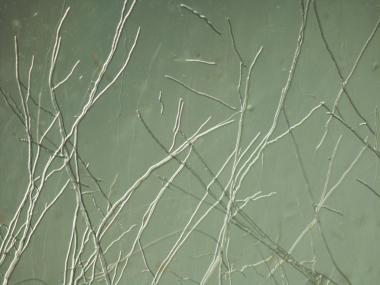

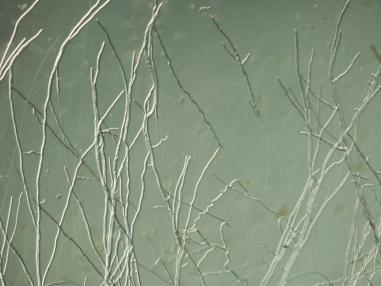

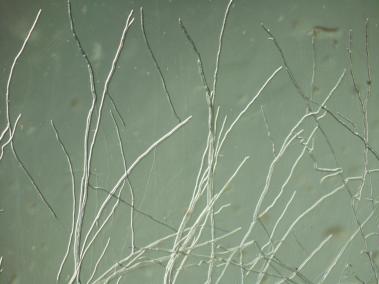

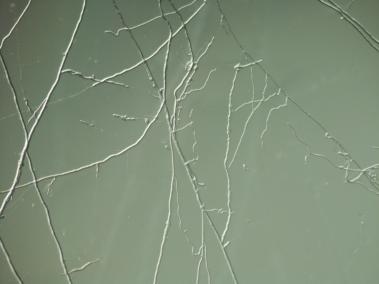

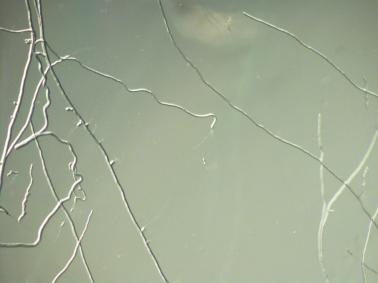

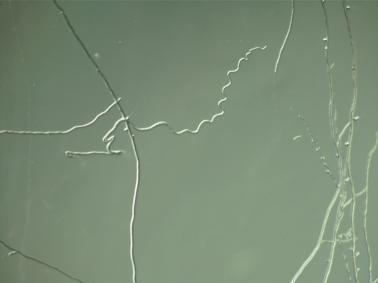

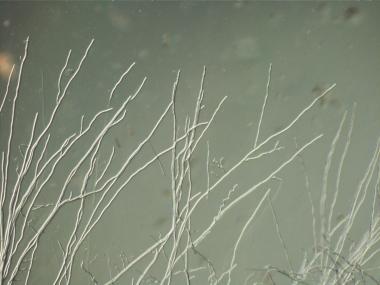

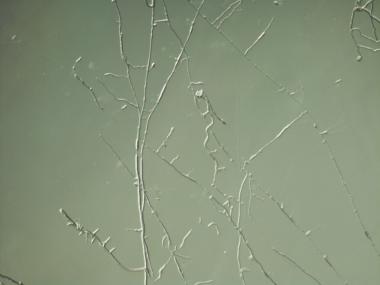

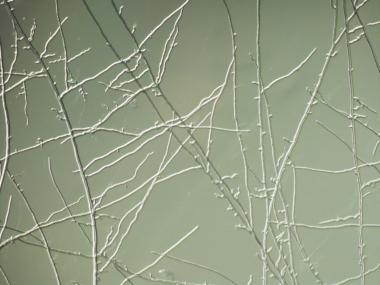

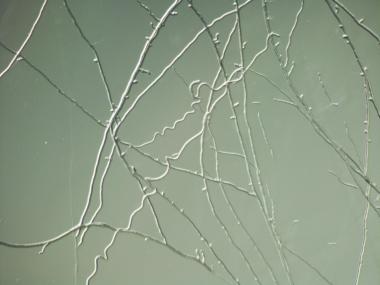

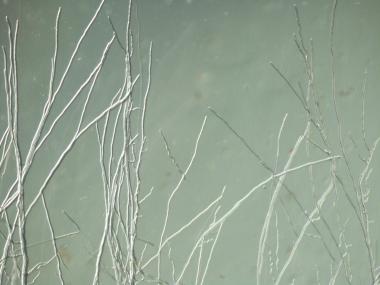

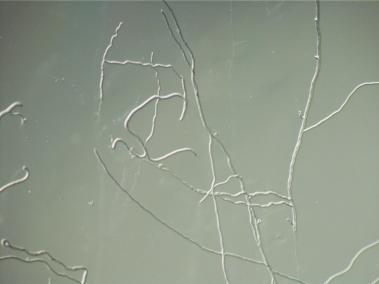

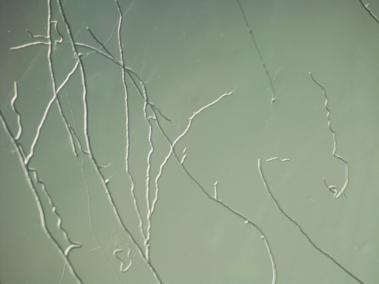

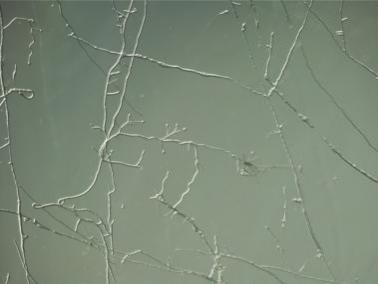


**Figure S4.** Mycelial morphology of four original *Fusarium* species and their FsoOLV1-infected derivatives (VI). Curved hyphae are indicated with red arrows. Scale bars=100 μm.
