# Supplementary material for: Identification of a novel hypovirulence-inducing ourmia-like mycovirus from Fusarium solani causing ginseng (Panax ginseng) root rot
Source: Front Microbiol. 2025 Jul 2;16:1609431. doi: 10.3389/fmicb.2025.1609431 (PMC12263584; doi:10.3389/fmicb.2025.1609431)
Supplement: Supplementary file 5 [file Table_5.docx]

Recipient strain VI 1 VI 2 VI 3


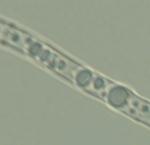

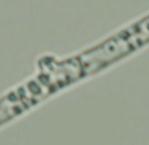

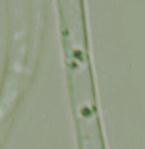

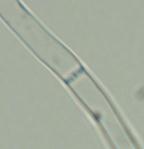

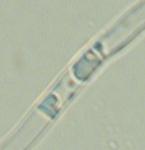

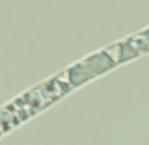

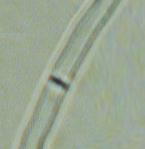

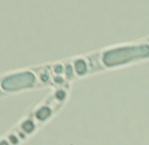

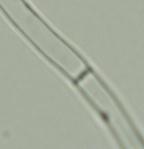

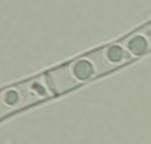

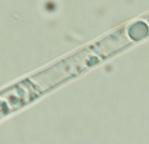

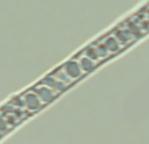

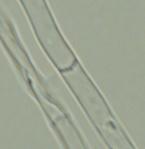

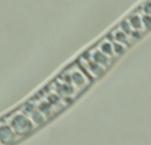

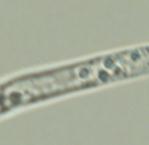

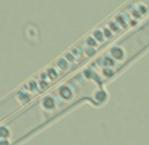


***F. oxysporum***

***F. verticillioides***

***F. proliferatum***

***F. solani***

**Figure S5.** Mycelial microscopic morphology of four original *Fusarium* species and their FsoOLV1-infected derivatives (VI). Scale bars=20 μm.
