# Supplementary material for: Identification of a novel hypovirulence-inducing ourmia-like mycovirus from Fusarium solani causing ginseng (Panax ginseng) root rot
Source: Front Microbiol. 2025 Jul 2;16:1609431. doi: 10.3389/fmicb.2025.1609431 (PMC12263584; doi:10.3389/fmicb.2025.1609431)
Supplement: Supplementary file 6 [file Table_6.docx]

**Table S1.** List of primers used in this study.

| Primer Name | Oligonucleotide sequence (5′-3′) | Usage |
| --- | --- | --- |
| ITS1 | TCCGTAGGTGAACCTGCGG | For the ribosomal RNA ITS region of *Fusarium* spp. |
| ITS4 | TCCTCCGCTTATTGATATGC |  |
| EF-1 | ATGGGTAAGGAAGACAAGAC | For the translation elongation factor 1 alpha of *Fusarium* spp. |
| EF-2 | GGARGTACCAGTSATCATGTT |  |
| 2-4F | ACTTGTCATTCAAGTTTTCCACG | Specific primers of FsoOLV1 |
| 2-4R | TTCCCAGAATTGATTCTGCGAATC |  |
| M13-47 | CGCCAGGGTTTTCCCAGTCACGAC | For terminal sequence verification |
| M13-48 | GAGCGGATAACAATTTCACACAGG |  |
| RACE1 | GGCAATTAACCCTCACTAAAG | For terminal sequence cloning |
| RACE2 | TCACTAAAGAATTCGATCGATC |  |
| RACE3 | CGATCGATCATGATGCAATGC |  |
| RACE-Oligo | 5'P-GCATTGCATCATGATCGATCGAATTCTTTAGTGAGGGTTAATTGCC-(NH2)-3' |  |
| 5’RACE2 | TACTGGGGAGGGTTTTACGG |  |
| 3’RACE2 | ACCGTTTTTGTGAAAGCCGA |  |
| 5’RACE3 | CGCGAACGTTTCCCAAGTTT |  |
| 3’RACE3 | ACGACTGGGATGACCGTAGA |  |
