# Supplementary material for: Identification of a novel hypovirulence-inducing ourmia-like mycovirus from Fusarium solani causing ginseng (Panax ginseng) root rot
Source: Front Microbiol. 2025 Jul 2;16:1609431. doi: 10.3389/fmicb.2025.1609431 (PMC12263584; doi:10.3389/fmicb.2025.1609431)
Supplement: Supplementary file 7 [file Table_7.docx]

**Table S2.** Biological characteristics of *Fusarium solani* strain SJH 2-4 and its derived virus-free strains 2-4 VF1-3.

| Strains | Growth rate (cm/day) | Spore yield (*10^6^/mL) | Germination rate (%) | 7 day Biomass (mg) |
| --- | --- | --- | --- | --- |
| SJH 2-4 | 0.89±0.01b | 15.90±0.49c | 59.71±6.09b | 19.50±0.71c |
| 2-4 VF1 | 1.12±0.01a | 22.25±0.35a | 86.65±3.27a | 33.50±0.71b |
| 2-4 VF2 | 1.11±0.01a | 20.25±0.71b | 82.30±6.91a | 41.50±2.12a |
| 2-4 VF3 | 1.09±0.01a | 19.38±0.18b | 77.47±6.08a | 46.00±1.41a |
