# Supplementary material for: Identification of a novel hypovirulence-inducing ourmia-like mycovirus from Fusarium solani causing ginseng (Panax ginseng) root rot
Source: Front Microbiol. 2025 Jul 2;16:1609431. doi: 10.3389/fmicb.2025.1609431 (PMC12263584; doi:10.3389/fmicb.2025.1609431)
Supplement: Supplementary file 8 [file Table_8.docx]

| **Table S3.** Biological characteristics of four *Fusarium* species before and after FsoOLV1 transfection. | | | | |
| --- | --- | --- | --- | --- |
| Strains | Growth rate (cm/day) | Spore yield (*10^6^/mL) | Germination rate (%) | 7 day Biomass (mg) |
| *F. oxysporum* | 1.22±0.01a | 11.05±1.95a | 98.67±0.58a | 49.33±1.53a |
| FoVI1 | 1.02±0.02b | 7.83±0.25b | 76.26±0.90b | 42.33±2.52bc |
| FoVI2 | 0.96±0.01c | 6.30±0.26bc | 72.66±1.02c | 47.00±4.58ab |
| FoVI3 | 0.81±0.02d | 4.23±0.43c | 70.62±0.88d | 39.67±1.53c |
| *F. verticillioides* | 1.09±0.01a | 27.07±4.80a | 73.15±1.02a | 47.33±2.08a |
| FvVI1 | 0.91±0.01b | 17.83±0.38b | 55.14±1.32b | 36.67±2.08b |
| FvVI2 | 0.88±0.01c | 10.82±0.83c | 48.22±0.83c | 35.67±1.53b |
| FvVI3 | 0.94±0.01b | 18.58±0.38b | 46.59±0.67c | 38.00±2.65b |
| *F. proliferatum* | 1.20±0.02a | 35.83±0.63a | 68.56±1.71a | 59.33±11.93a |
| FpVI1 | 0.96±0.02b | 31.67±0.55c | 43.57±2.82c | 27.33±10.69b |
| FpVI2 | 0.99±0.02b | 34.17±1.04b | 49.33±2.08b | 40.67±12.86ab |
| FpVI3 | 0.97±0.04b | 28.25±0.25d | 41.95±0.66c | 41.33±6.66ab |
| *F. solani* | 1.20±0.01a | 18.27±0.65a | 66.71±11.87a | 72.00±1.00a |
| FsVI1 | 0.96±0.01b | 9.23±0.06b | 50.92±0.69b | 50.67±2.52c |
| FsVI2 | 0.96±0.01b | 9.48±0.08b | 52.22±1.13b | 65.33±3.06b |
| FsVI3 | 0.98±0.02b | 9.37±0.08b | 46.92±1.14b | 61.33±1.53b |
